# Supplementary material for: Meta-Analysis and Gene Set Enrichment Relative to ER Status Reveal Elevated Activity of MYC and E2F in the “Basal” Breast Cancer Subgroup
Source: PLoS One. 2009 Mar 9;4(3):e4710. doi: 10.1371/journal.pone.0004710 (PMC2650420; doi:10.1371/journal.pone.0004710)
Supplement: Dataset S1 — GSEA results from first screen. The file contains a zipped archive of the HTML results of the second GSEA screen. Reports for enriched or depleted datasets may be accessed from the “index.html” link within the my_analysis.GseaPreranked.1219975950625.rpt.zip ZIP archive. Enrichment plots are available for each of the 50 most enriched and depleted gene sets. (13.32 MB ZIP) [file pone.0004710.s001.zip › my_analysis.GseaPreranked.1219975950625.rpt.zip!//ADIP_DIFF_CLUSTER1.html]

Details for gene set ADIP\_DIFF\_CLUSTER1[GSEA]

|  || Dataset | zSco.ERneg.to.ERpos\_collapsed |
| Phenotype | NoPhenotypeAvailable |
| Upregulated in class | na\_neg |
| GeneSet | ADIP\_DIFF\_CLUSTER1 |
| Enrichment Score (ES) | -0.43012193 |
| Normalized Enrichment Score (NES) | -1.7813137 |
| Nominal p-value | 0.0 |
| FDR q-value | 0.04164215 |
| FWER p-Value | 0.824 |
Table: GSEA Results Summary

  

Fig 1: Enrichment plot: ADIP\_DIFF\_CLUSTER1      
 Profile of the Running ES Score & Positions of GeneSet Members on the Rank Ordered List

  

| PROBE | GENE SYMBOL | GENE\_TITLE | RANK IN GENE LIST | RANK METRIC SCORE | RUNNING ES | CORE ENRICHMENT || 1 | PDGFRA |  |  | 556 | 4.224 | -0.0036 | No |
| 2 | ADFP |  |  | 1342 | 3.090 | -0.0347 | No |
| 3 | SERPINB9 |  |  | 1974 | 2.553 | -0.0591 | No |
| 4 | GAS1 |  |  | 1982 | 2.544 | -0.0366 | No |
| 5 | GAS2 |  |  | 2222 | 2.372 | -0.0331 | No |
| 6 | NOV |  |  | 2228 | 2.368 | -0.0120 | No |
| 7 | ARD1A |  |  | 2246 | 2.354 | 0.0081 | No |
| 8 | CSF1 |  |  | 2414 | 2.245 | 0.0159 | No |
| 9 | MAP4 |  |  | 2611 | 2.140 | 0.0205 | No |
| 10 | TLN1 |  |  | 2877 | 1.998 | 0.0187 | No |
| 11 | DDEF1 |  |  | 3272 | 1.805 | 0.0053 | No |
| 12 | RUNX1T1 |  |  | 3490 | 1.714 | 0.0045 | No |
| 13 | LPL |  |  | 3611 | 1.658 | 0.0105 | No |
| 14 | SPP1 |  |  | 4426 | 1.346 | -0.0386 | No |
| 15 | TPM1 |  |  | 4482 | 1.324 | -0.0308 | No |
| 16 | F3 |  |  | 5288 | 1.053 | -0.0819 | No |
| 17 | IL11RA |  |  | 5605 | 0.943 | -0.0972 | No |
| 18 | PSMB10 |  |  | 6383 | 0.691 | -0.1495 | No |
| 19 | MYL9 |  |  | 6475 | 0.659 | -0.1503 | No |
| 20 | C5ORF13 |  |  | 6512 | 0.645 | -0.1472 | No |
| 21 | IDH1 |  |  | 6741 | 0.576 | -0.1592 | No |
| 22 | ISG15 |  |  | 6857 | 0.533 | -0.1630 | No |
| 23 | RALGDS |  |  | 6996 | 0.489 | -0.1690 | No |
| 24 | ACTA2 |  |  | 7342 | 0.369 | -0.1916 | No |
| 25 | APBB1IP |  |  | 7591 | 0.272 | -0.2078 | No |
| 26 | FLOT1 |  |  | 8302 | 0.001 | -0.2614 | No |
| 27 | GADD45A |  |  | 8982 | -0.305 | -0.3098 | No |
| 28 | FIGF |  |  | 9109 | -0.366 | -0.3160 | No |
| 29 | COL11A1 |  |  | 9215 | -0.412 | -0.3201 | No |
| 30 | IGF1 |  |  | 9639 | -0.637 | -0.3462 | No |
| 31 | MXD4 |  |  | 9642 | -0.639 | -0.3406 | No |
| 32 | DAB2 |  |  | 9682 | -0.660 | -0.3375 | No |
| 33 | RAB11FIP5 |  |  | 9708 | -0.673 | -0.3333 | No |
| 34 | DDIT3 |  |  | 9789 | -0.718 | -0.3328 | No |
| 35 | PCCB |  |  | 9933 | -0.801 | -0.3364 | No |
| 36 | VEGFC |  |  | 10580 | -1.225 | -0.3739 | No |
| 37 | MGP |  |  | 10790 | -1.382 | -0.3771 | No |
| 38 | RASL11B |  |  | 11074 | -1.643 | -0.3836 | No |
| 39 | FZD2 |  |  | 11165 | -1.716 | -0.3748 | No |
| 40 | TRIB3 |  |  | 11900 | -2.438 | -0.4080 | Yes |
| 41 | THBS2 |  |  | 11978 | -2.536 | -0.3908 | Yes |
| 42 | CTSK |  |  | 12011 | -2.574 | -0.3699 | Yes |
| 43 | SERF2 |  |  | 12297 | -2.964 | -0.3645 | Yes |
| 44 | SORD |  |  | 12298 | -2.964 | -0.3376 | Yes |
| 45 | TIMP3 |  |  | 12387 | -3.085 | -0.3162 | Yes |
| 46 | OGN |  |  | 12509 | -3.275 | -0.2956 | Yes |
| 47 | AES |  |  | 12534 | -3.313 | -0.2674 | Yes |
| 48 | PCK2 |  |  | 12617 | -3.462 | -0.2422 | Yes |
| 49 | KLF2 |  |  | 12701 | -3.625 | -0.2155 | Yes |
| 50 | TGFB3 |  |  | 12734 | -3.702 | -0.1844 | Yes |
| 51 | PDGFRB |  |  | 12886 | -4.104 | -0.1585 | Yes |
| 52 | PLAT |  |  | 13127 | -4.926 | -0.1319 | Yes |
| 53 | CCNG2 |  |  | 13157 | -5.088 | -0.0879 | Yes |
| 54 | FAH |  |  | 13211 | -5.503 | -0.0420 | Yes |
| 55 | CYB5R1 |  |  | 13214 | -5.529 | 0.0080 | Yes |
Table: GSEA details [plain text format]

  

Fig 2: ADIP\_DIFF\_CLUSTER1: Random ES distribution      
 Gene set null distribution of ES for **ADIP\_DIFF\_CLUSTER1**
